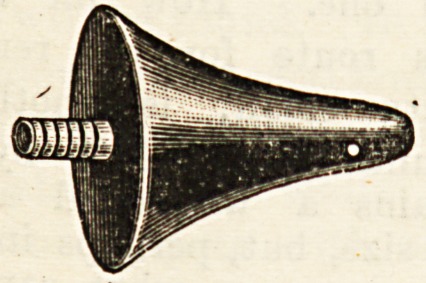# New Appliances and Things Medical

**Published:** 1900-05-19

**Authors:** 


### NEW APPLIANCES AND THINGS MEDICAL.

**[We shall be glad to receive, at our Office, 28 & 29, Southampton Street, Strand, London, W.C., from the manufacturers, specimens of all new preparations and appliances which may be brought out from time to time.]**

### A NEW FORM OF ENEMA NOZZLE.

(Messrs. Sumner and Co., Lord Street, Liverpool.)

This ingenious invention only requires to be known to be widely appreciated. The difficulty of inducing patients to retain enemas is a constantly recurring problem which Dr.

Alexander Duke appears to have solved, and we congratulate him upon the manner in which he has done it. The contrivance, of which we give an illustration, is conical in shape and wide at the base. It is passed firmly into the bowel, the injected fluid being effectually retained by virtue of its wedge-shaped end plugging the anus and forcing the sphincter to embrace it closely. It will be found especially useful in the case of children whose struggles to expel the tube render the administration of an enema a difficult matter. By the use of this form of nozzle the operation is easily enough performed. In cases where it is found necessary to give saline injections, now so often ordered after severe hæmorrhage or shock following operations, it will be found most valuable, as the shape of the nozzle renders it much less likely for any fæcal matter to adhere to the tube on its being withdrawn, consequently it can easily be kept clean. The makers keep the nozzle in two sizes for adults and children.

## Figures and Tables

**Figure f1:**